# A Framework for Analysis of Abortive Colony Size Distributions Using a Model of Branching Processes in Irradiated Normal Human Fibroblasts

**DOI:** 10.1371/journal.pone.0070291

**Published:** 2013-07-23

**Authors:** Tetsuya Sakashita, Nobuyuki Hamada, Isao Kawaguchi, Noriyuki B. Ouchi, Takamitsu Hara, Yasuhiko Kobayashi, Kimiaki Saito

**Affiliations:** 1 Microbeam Radiation Biology Group, Japan Atomic Energy Agency (JAEA), Gunma, Japan; 2 Radiation Safety Research Center, Nuclear Technology Research Laboratory, Central Research Institute of Electric Power Industry (CRIEPI), Tokyo, Japan; 3 Regulatory Science Research Program, Research Center for Radiation Protection, National Institute of Radiological Sciences (NIRS), Chiba, Japan; 4 Radiation Effect Analysis Group, JAEA, Ibaraki, Japan; 5 Advanced Clinical Research Center, Fukushima Global Medical Science Center, Fukushima Medical University, Fukushima, Japan; 6 Fukushima Environmental Safety Center, JAEA, Tokyo, Japan; Universitat Rovira i Virgili, Spain

## Abstract

**Background:**

Clonogenicity gives important information about the cellular reproductive potential following ionizing irradiation, but an abortive colony that fails to continue to grow remains poorly characterized. It was recently reported that the fraction of abortive colonies increases with increasing dose. Thus, we set out to investigate the production kinetics of abortive colonies using a model of branching processes.

**Methodology/Principal Findings:**

We firstly plotted the experimentally determined colony size distribution of abortive colonies in irradiated normal human fibroblasts, and found the linear relationship on the log-linear or log-log plot. By applying the simple model of branching processes to the linear relationship, we found the persistent reproductive cell death (RCD) over several generations following irradiation. To verify the estimated probability of RCD, abortive colony size distribution (≤15 cells) and the surviving fraction were simulated by the Monte Carlo computational approach for colony expansion. Parameters estimated from the log-log fit demonstrated the good performance in both simulations than those from the log-linear fit. Radiation-induced RCD, i.e. excess probability, lasted over 16 generations and mainly consisted of two components in the early (<3 generations) and late phases. Intriguingly, the survival curve was sensitive to the excess probability over 5 generations, whereas abortive colony size distribution was robust against it. These results suggest that, whereas short-term RCD is critical to the abortive colony size distribution, long-lasting RCD is important for the dose response of the surviving fraction.

**Conclusions/Significance:**

Our present model provides a single framework for understanding the behavior of primary cell colonies in culture following irradiation.

## Introduction

Clonogenicity, i.e., the ability of a cell to form a colony gives important information about the cellular reproductive potential that changes in response to various internal and external stimuli. The colony formation assay has been widely used over the past half a century to determine cell survival, especially in the field of toxicology and radiation biology [1], [2], [3], [4], where “clonogenic” colonies each containing 50 cells or more are generally considered as survivors. On the other hand, colonies of less than 50 cells that are unable to continue to grow within several generations after the insults are termed “abortive” colonies [1], but their growth kinetics remains poorly understood. Given mounting evidence for non-targeted effects of ionizing radiation (IR) such as genomic instability and bystander effects [5], [6], [7], the analysis of abortive colonies would be important and should make the colony formation assay more informative. In this regard, we previously reported that, in IR-exposed normal human fibroblasts, the fraction of abortive colonies increases with increasing dose in contrast to the case for clonogenic colonies [8], suggesting a systematic rule in the production of abortive colonies. With more detailed biological data sets of abortive colonies and a simple branching process model analysis, we here set out to address how an abortive colony is formed following IR. In addition, to test the compatibility of the branching process analysis in clonogenic colonies, we compared the experimentally determined clonogenic colonies to simulated ones by the two-dimensional Monte Carlo method based on the stochastic branching processes. The present study provides a new framework for analysis of colony size distribution in abortive and clonogenic colonies.

## Materials and Methods

### Cell Culture, Irradiation and Colony Formation Assay

AG01522D primary normal human diploid foreskin fibroblasts were purchased from the Coriell Cell Repositories at the Coriell Institute for Medical Research (Camden, NJ), and used for all experiments. Cell cultures, irradiation with ^60^Co γ-rays, colony formation assay were conducted as previously described [8]. Colonies with 2–49 cells are referred hereinafter to as abortive colonies, and those with 50 cells or more as clonogenic colonies. Colonies derived from AG01522D cells are a flat monolayer [9], and the mean plating efficiency (i.e., the number of clonogenic colonies divided by that of plated cells) was 28.8%. We counted colonies containing 2 or more cells. Summarized data for the analysis of colonies and surviving fraction has been previously reported [8], and its original detailed data sets were used in this study. Growth curve (Figure S1) was obtained by counting cell numbers in duplicate at each day up to 11 days to prepare confluent cultures after plating, and experiments were repeated three times.

### Cell Lineage and Branching Process

**Figure 1:**
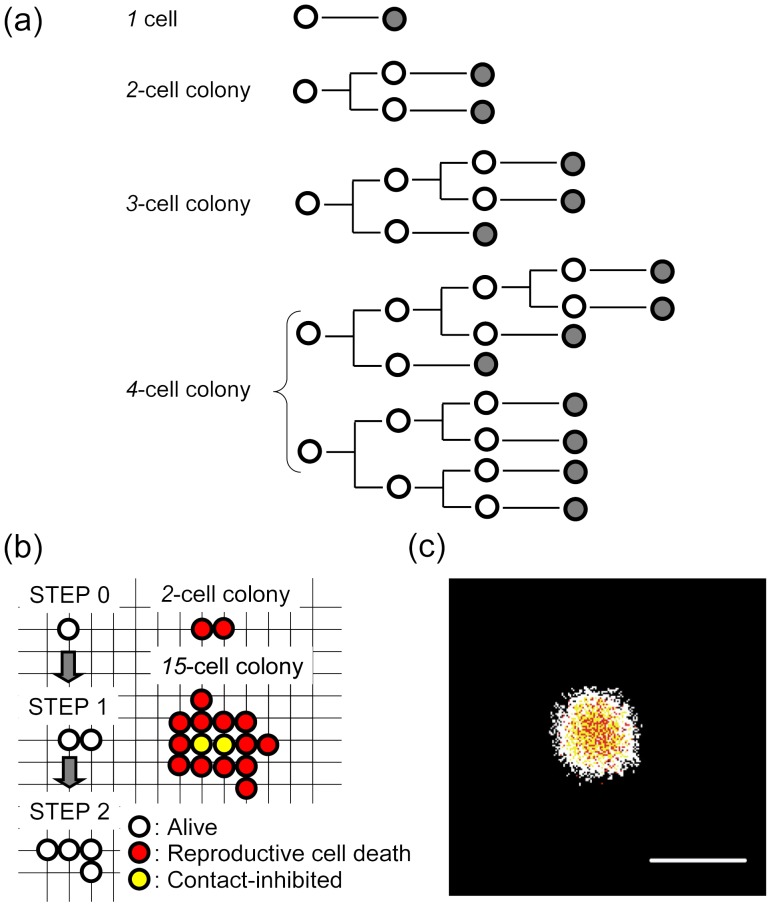
Outline of the branching dynamics model. (a) Example cell lineage of abortive colonies. (b) Illustration of colony formation in the two-dimensional colony expansion using the Monte Carlo method. Firstly, a cell was located on the grid point of which size was set as 31.6 µm×31.6 µm (a cell size of 1,000 µm^2^). Only when a cell has a free space in adjacent grid points, the cell can divide or move. In contrast, a cell without adjacent space cannot proliferate (by contact inhibition). (c) An example image of a simulated colony. Scale bar, 2,000 µm.

At the recurring branch of the cell lineage in cells exposed to ^60^Co γ-rays, a cell should undergo reproductive cell death (RCD) or proliferation, and forms an abortive colony with a complicated cell lineage expressed as a branch tree (Figure 1a). To analyze the production kinetics of abortive colonies, we introduced *P_1_* (probability of RCD), *P_2_* (probability of proliferation, i.e., equal to 1 minus *P_1_*) at each branch point, and the *f_n_* (occurrence frequency of abortive colonies with *n* cells) was calculated as follows with Equation 1:
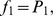


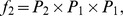


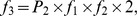


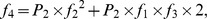


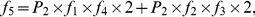
(1)

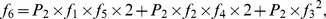


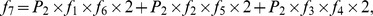


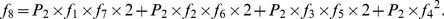



In the simple mock examination, *P_1_* was fixed for all generations, and we evaluated a relationship between *f_n_* and colony size in the branch dynamics. The frequency of colonies with 1 cell was estimated from that of 2-cell colonies, assuming that the similar probability of RCD for 1- and 2-cell colonies. The word “frequency for all colonies” refers herein to the distribution of all colonies (abortive including 1-cell colony plus clonogenic), and the word “frequency” was used for the distribution of colonies with 2 or more cells. Moreover, we evaluated *P_1_* (*g*) with a flexible change at generation *g* from the regression curve with 95% confidence limit using experimental data sets and the frequency of 1-cell colony estimated by the stochastic branching process.

### Simulation Model and Calculation

To verify the stochastic branching analysis with the estimation of the frequency of 1-cell colony, we compared the observed clonogenic colonies and simulated ones, by using the two-dimensional Monte Carlo method based on the stochastic branching processes. A clonogenic colony containing many cells has various intercellular restrictions like contact inhibition, making it difficult to estimate probabilities of all events even with careful analytical evaluations. Monte Carlo simulation is a better alternative for such complicated and repetitive phenomena. We developed the simulation model considering two-dimensional colony expansion using the Monte Carlo method, because colonies derived from AG01522D fibroblasts form a flat monolayer. Firstly, a cell was located on the grid point of the two-dimensional calculation grid of which size was set as 31.6 µm×31.6 µm (Figure 1b). A cell size was set as 1,000 µm^2^. Only when a cell has a free space in adjacent 8 grid points, the cell can divide into two cells or move to a free space. On the other hand, cells without adjacent space cannot proliferate, i.e. by contact inhibition. To test if the contact inhibition designed to occur in our model actually does occur, we conducted the simulation for growth curve (Figure S1). We used the experimentally determined doubling time of 20 h [4], and calculated the fate of irradiated cells at each doubling according to *P_1_* (*g*). Cells were allowed to form a clonogenic colony for about 13 days (≤16 divisions) after IR, as was done in experiments [8]. One simulation consists of 10,000 times of the calculation based on the 1-cell inoculation into the two-dimensional calculation grid. Five simulation sets were carried out for each calculation condition, and total inoculated cell numbers were 50,000. Using a Windows PC with a core-i7 CPU, it took 10 min –6 h to execute one simulation.

### Platform

To perform the repetitive and complicated calculations, we developed the individual- cell-based model, using the platform of the NetLogo software [10] that is freely downloadable at http://ccl.northwestern.edu/netlogo/. NetLogo is a java platform that has been used to analyze complex systems, and adopts the multi-agent modeling Logo languages.

### Statistical Analysis

The computer program (ORIGIN, MicroCal Software, Inc., MA, USA) was used for the non-linear regression analysis and 95% confidence limit on the colony size distribution. A BETTER fit model was selected with the Akaike Information Criterion (AIC) with log transformed data except for the estimated frequency of 1-cell colony. Statistical comparisons between experimental data and model estimation were made using the Chi square test, and P-values were corrected using Bonferroni correction method.

## Results

### Experimental Colony Size Distribution of Abortive Clones

**Figure 2:**
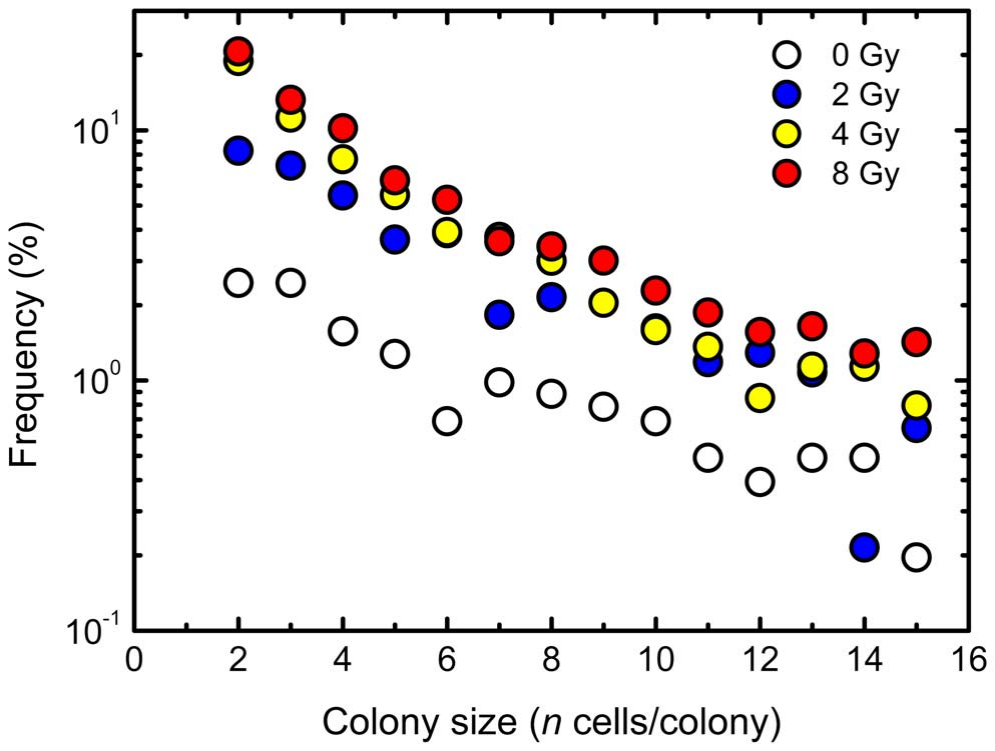
Empirical colony size distribution. Circles indicate the experimentally determined data at 0, 2, 4 and 8 Gy of ^60^Co γ-rays. “Frequency” represents the frequency of a colony with *n* cells in all abortive and clonogenic colonies.

When the experimentally obtained data were plotted on the log-linear graph, there was a linear relation between the frequency of abortive colonies derived from non-irradiated cells and the number of cells per colony, suggestive of an exponential relation (Figure 2). Intriguingly, this was also indeed the case for abortive colonies derived from irradiated cells (Figure 2).

### Colony Size Distribution Based on the Branching Processes

**Figure 3:**
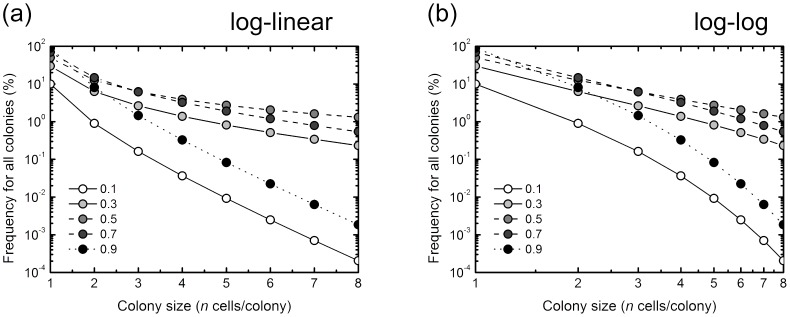
Mock examination of the branching dynamics with a probability of reproductive cell death (*P_1_*) on the log-linear plot (a) and log-log plot (b). The same *P_1_* value at all branch points of the cell lineage was assumed, and *Freq* (occurrence frequency of abortive colony with *n* cells) was calculated. *P_1_* ranged from 0.1 to 0.9.

Cell division system is generally a branching process, where a cell divides into two daughter cells except for a germ cell. Before interpreting experimental data sets in a branching process, we firstly noticed that the colony size distribution greatly depends on the specific condition of branch process, i.e. *P_1_*, and that colony size distribution shows a steep slope between 1-cell and 2-cell colony sizes on the log-linear plot. In this mock examination, we assumed the same *P_1_* value at all branch points of the cell lineage (Figure 1a and Equation 1), and the *f* value was calculated with Equation 1. *P_1_* ranged from 0.1 to 0.9. The simple mock examination demonstrated that the linear relationship in colonies with 2 or more cells on the log-linear plot is established by *P_1_* of 0.3 to 0.7, but not 0.1 and 0.9 (Figure 3a). This shows that a linear relationship in the log-linear plot generated by the branching dynamics needs a suitable *P_1_* value. However, there was a steep slope between 1-cell and 2-cell colony sizes. The result indicates that, using the data sets for size distribution of colonies with 2 or more cells, a linear regression analysis on the log-linear plot underestimates a frequency of 1-cell colony that follows a branching process. On the other hand, the colony size distribution on the log-log plot demonstrated the linear relationship even at the 1 cell colony size, suggesting that a linear regression analysis on the log-log plot is adequate for the colony size distribution produced by the branch process.

### Regression Analysis for the Size Distribution of Experimentally Observed Abortive Colonies

**Figure 4:**
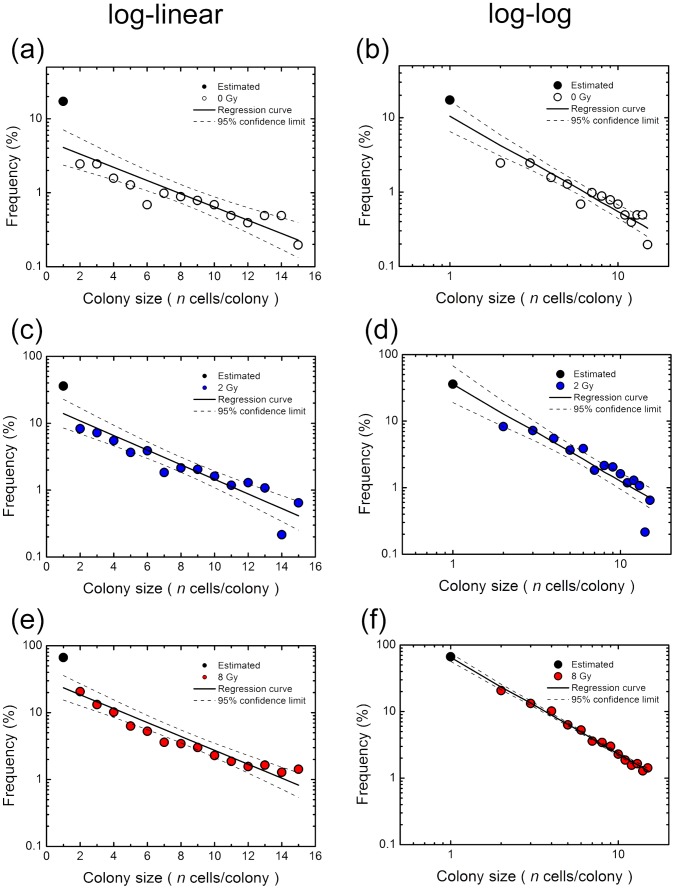
Regression analysis of experimental colony size distribution of abortive colonies at 0, 2 and 8 Gy. Two regression analyses on the log-linear (a, c and e) and log-log (b, d and f) plots were tested. Here, Equation 1 was used to estimate the frequency of 1-cell colony size (solid circle) from that of 2-cell colonies, assuming the similar probability of reproductive cell death for 1- and 2-cell colonies. The straight lines show the linear regression curves, and the dotted lines show the curves of a 95% confidence limit. Table 1 lists the numerical values of parameters obtained from the regression analysis of the data set.

When the experimentally obtained data was plotted on the log-linear graph, there was a linear relation between the frequency of abortive colonies derived from non-irradiated cells and the number of cells per colony, suggestive of an exponential relation (Figure 2). Intriguingly, the same held true for abortive colonies derived from irradiated cells (Figure 2). However, the mock examination of a branching process model showed a steep slope in the log-linear plot between 1-cell and 2-cell colony sizes, and demonstrated the effectiveness of the linear regression analysis on the log-log plot (Figure 3a and 3b). Therefore, we tested the two regression analyses on the log-linear and log-log plots. Here, we do not know actual frequency of 1-cell colony size, but estimated it using the Equation 1, predicated on the frequency of 2-cell colonies assuming the similar probability of RCD for 1- and 2-cell colonies. The linear regression analysis with a 95% confidence limit on the log-log plot demonstrated A BETTER fit to the data sets than that of the log-linear plot (Figure 4a–4f). Table 1 lists the numerical values of parameters obtained from the regression analysis to the data set. As shown in Table 1, *R^2^* values of the resulting regression curves of the log-log plot were greater than those of the log-linear plot as presented in Figure 4a–4f. In addition, we examined the AIC for two methods to fit the observed data, except for the estimated frequency of 1-cell colony. According to the resulting AIC values listed in Table 2, the log-log fitting model was determined as A BETTER fit model for 4 Gy and 8 Gy, and the log-linear fitting model was determined as A BETTER fit model for 0 Gy and 2 Gy. These results suggest that the linear regression on the log-log plot is a good choice for the branching process analysis of the abortive colony size distribution.

**Table 1:** Parameter values obtained from the log-linear (log y = a x+b) or log-log (log y = a log x+b) fits to the experimental data set.

Dose (Gy)	log y = a x+b			log y = a log x+b		
	a	b	*R^2^*	a	b	*R^2^*
0	−0.089	0.70	0.77	−1.28	1.02	0.91
2	−0.11	1.25	0.86	−1.45	1.55	0.88
4	−0.12	1.46	0.90	−1.60	1.82	0.99
8	−0.10	1.48	0.89	−1.45	1.81	0.99

*R^2^* denotes the correlation coefficient square.

**Table 2:** Akaike Information Criteria (AIC) values for two methods to fit the data, except for the estimated frequency of 1-cell colony.

AIC				
Method	Dose (Gy)			
	0	2	4	8
Log-linear	−18.410	−7.746	−19.023	−21.108
Log-log	−16.509	−2.361	−30.171	−44.430

Two methods examined are regression analyses on the log-linear and log-log plots.

### Normal Probability and IR-induced Excess Probability of RCD over Several Generations

**Figure 5:**
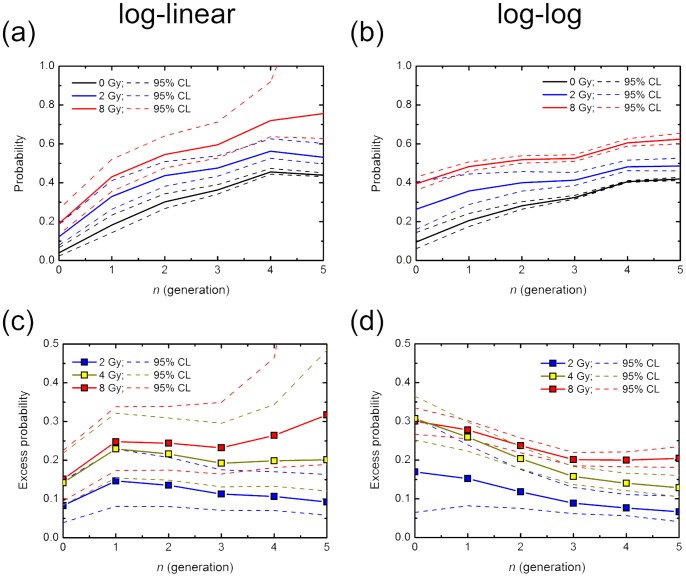
Reproductive cell death probability *P_1_* (0, 2 and 8 Gy) and the excess *P_1_* (2, 4 and 8 Gy) at 0 to 5 generations. *P_1_* was calculated with the simultaneous equations Equation 1, using the regression curves in Figure 4a–4f. IR-induced excess of *P_1_* was estimated by *P_1_* of abortive colonies derived from non-irradiated cells minus that from irradiated ones. Dotted lines show the 95% confidence limit.

The same *P_1_* values at all branch points (generations) of the cell lineage may not be realistic. Although the exact *P_1_* values are still unknown, the average *P_1_* at each generation can be deduced from the experimental data sets (Figure 4). We extended the simple model on branching dynamics (Figure 3) to the case in which *P_1_* at *g* generation expressed as *P_1_* (*g*) was flexible. Using the log-linear and log-log regression curves of the experimental data sets (Figure 4a–f), *P_1_* (*g* = 0 to 5) were calculated with simultaneous equations (Equation 1). Frequency of colonies with 16 cells or more was uncertain because of statistical fluctuations posed by the limited data sets, so that *P_1_* (*g* = 5) was fraught with uncertainty of the estimation. *P_1_* (*g*) of non-irradiated cells deduced from both regression curves increased with generations, reaching 0.4 (Figure 5a and 5b). On the other hand, *P_1_* (*g*) of irradiated cells also increased with generations, but the increments of the probability of the log-log plot were smaller than that of the log-linear plot (Figure 5a and 5b). Such difference that seems most likely to be attributable to the underestimates of the frequency of 1-cell colony size resulted in the difference in the pattern of the excess probabilities (Figure 5c and 5d). In addition, a 95% confidence limit in the log-linear analysis was greater than that in the log-log plot analysis (Figure 5a–5d). It is likely that the probability and the excess probability in the log-log plot analysis are closer to the true values than those in the log-linear plot analysis, and those in the log-log plot analysis were used in the analyses described below. Despite the difference in the patterns of the probability and IR-induced excess probability of RCD between them, these results suggest that IR-induced excess probability lasts over several generations. This finding provides evidence for non-targeted radiation effects like genomic instability.

### Computational Simulation of Abortive and Clonogenic Colonies Arising from Non-irradiated Cells

**Figure 6:**
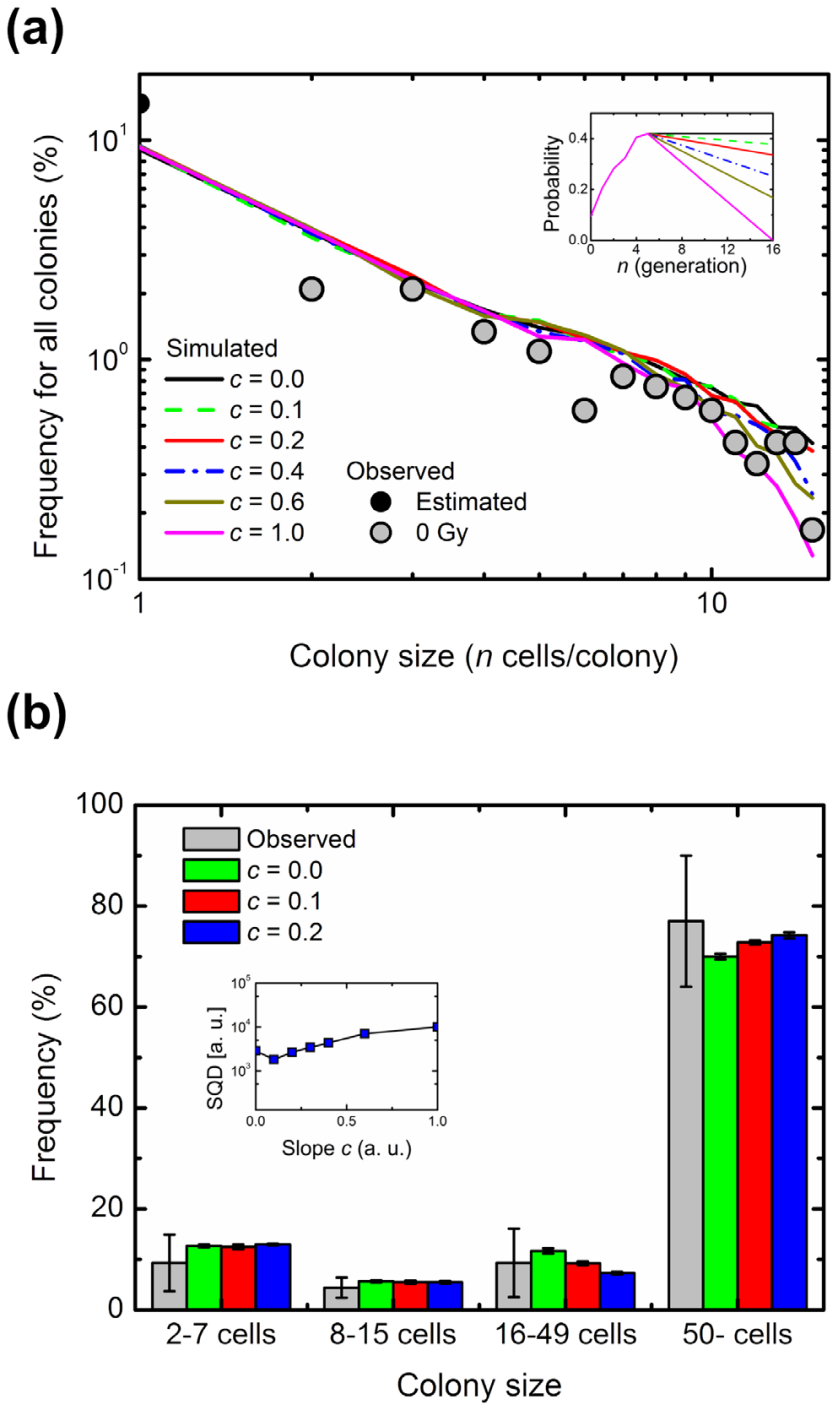
Computational simulation of non-irradiated abortive and clonogenic colonies using the parameters of the log-log fit. (a) Observed and simulated abortive colony size distribution. Gray circles show the observed colony size, and lines show the simulated colony size distribution with the slope *c* ranging from 0.0 to 1.0. A solid circle is the estimated frequency of 1-cell colony. Several patterns of *P_1_* with the slope *c* are shown in the inset. (b) Comparison of simulated results and experimental data sets including the frequency of colonies with ≥16 cells. Error bars indicate the standard deviations. The inset demonstrates the square of the difference (SQD) between simulated and observed frequencies, calculated as Σ{(*f*_simulated_ – *f*_experimental_ )/*f*_experimental_ * 100}^2^, where *f* is the percentage of colonies with *n* cells, each of which was classified into 2–7, 8–15, 16–49 or ≥50 cells. a.u., arbitrary units.

A simple branching process may provide important information in the colony-formation kinetics of abortive colonies. There are, however, an assumption in the frequency of 1-cell colony size and a problem that the fit is only over a small range of colony sizes. It is therefore necessary to test over a broader range of colony sizes for the estimation in the branching process analysis. Here, we addressed this by using the experimental data sets for the frequency of colonies with ≥16 cells [8]. A clonogenic colony with ≥50 cells consists of cells with various generations as a consequence of persistent RCD. Also, the possibility of the contact inhibition cannot be ruled out in the case of a clonogenic colony. Thus, to analyze *in silico* clonogenic colonies based on the branching dynamics, we employed a simulation model considering two-dimensional colony expansion by the Monte Carlo method, as shown in Figure 1c. We confirmed that the simulation model could simulate the small abortive colony size distribution with fixed *P_1_* values (Figure S2). Here, we did not have enough data for the frequency of colonies with ≥16 cells that should have undergone a minimum of 4 divisions. Six cell divisions (i.e., 5 generations) generally result in production of a colony containing 64 cells, which is classified as a survivor. Therefore, for this simulation, we used the *P_1_* (*g* ≤*5*) value determined by the regression curves (Figures 4a–4f and 5a–5d), and the simple linear model for *P_1_* (*g* ≥*6*) was assumed in which *P_1_* (*g* = *16*) was set as (1– *c*) multiplied by *P_1_* (*5*), and the values between them were interpolated (insets in Figures 6a and S3a).

Simulated results using the probability deduced from the log-log plot analysis were well consistent with the observed patterns in the size of abortive colonies derived from non-irradiated cells, and the value of *c* changed the simulated frequency of colonies with ≥10 cells (Figure 6a). We used AIC to evaluate the appropriate model with the parameter of *c*. As is evident from Table 3, the lowest AIC value was obtained at *c* = 0.4. On the other hand, there was a systematic discrepancy between the observed colony size distribution and that resulting from the log-linear analysis (Figure S3a). Furthermore, we compared the simulated results with experimental data sets for frequency of colonies with ≥16 cells [8]. For this, the square of difference (SQD) between simulated and experimentally determined frequencies was introduced as Σ{(*f*_simulated_ – *f*_experimental_ )/*f*_experimental_×100}^2^, where *f* is the percentage of colonies with *n* cells that was classified into 2–7, 8–15, 16–49 and ≥50 cells as shown in Figure 6b. Simulated results (*c* = 0.0 to 0.2) using the probability deduced from the log-log plot analysis in frequency of 16–49 cells and clonogenic colonies were nearly identical to experimentally determined data and within the standard deviation (Figure 6b). The least SQD was demonstrated in *c* of 0.1 (Figure 6b). There was one order of magnitude difference between SQD in the log-linear analysis and that in the log-log analysis (insets in Figures 6b and S3b). These results indicate that the two-dimensional colony expansion model using the probability deduced from the log-log plot analysis and the parameter of *c* in a range between 0.1 and 0.4 well simulated the abortive colony size distribution and the frequency of colonies with 16–49 cells and clonogenic colonies, whereas that from the log-linear plot analysis showed a systematic discrepancy and the large SQD.

**Table 3:** Alterations of AIC values as a function of *c* values.

c	0.0	0.1	0.2	0.3	0.4	0.6	1.0
AIC	6.017	3.212	4.360	1.627	−2.608	0.085	5.062

The parameter of *c* was used for the simple linear model for *P_1_* (*g* ≥6) where *P*_1_ (*g* = 16) was set as (1– *c*) multiplied by *P_1_* (5).

### Simulated Size Distribution of Abortive Colonies Derived from Irradiated Cells

**Figure 7:**
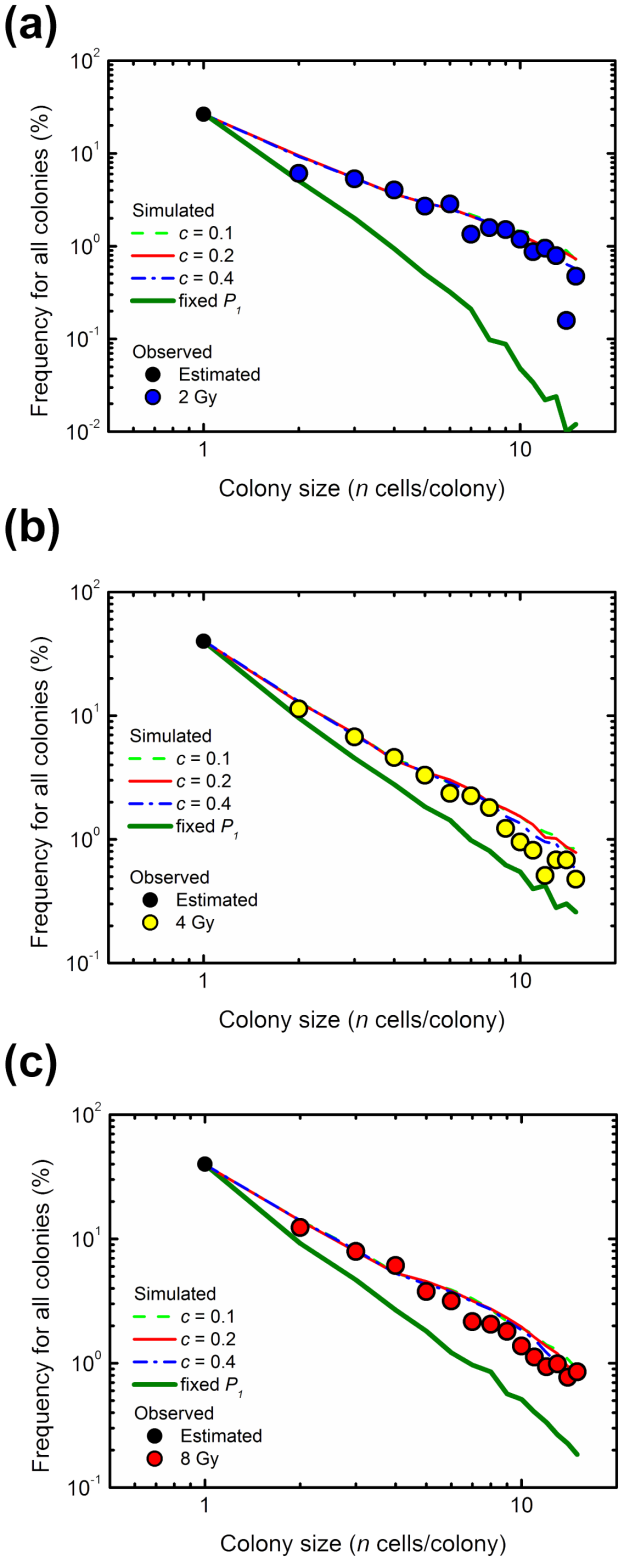
Simulated colony size distribution of irradiated abortive clones. The abortive colony size distributions at 2, 4 and 8 Gy are shown in panels (a), (b) and (c), respectively. Circles demonstrate the experimentally determined size of colonies, and lines represent the simulated colony size distribution with the specific slope *c* (0.1, 0.2 and 0.4) using the parameters of the log-log fit. The data of the fixed *P_1_* shows the result of the simulation using the *P_1_* (*g* = 0) at all generations.

There was a linear relationship of abortive colonies on the log-log plot in irradiated conditions (Figures 2 and 4a–4f). Therefore, to investigate whether IR-induced excess of *P_1_* (*g*) based on the branch dynamics can generate the linear relationship in the frequency of the abortive colony size on the log-log plot, we simulated abortive colony formation following IR using the probability of *P_1_* (*g*) (Figure 5b). Here, the decrease in the probability of *P_1_* (*g* ≥*6*) was assumed in the same way as *P_1_* (*g* ≥*6*) of abortive colonies derived from non-irradiated cells (Figure 6). As shown in Figure 7, our model using the parameter of the log-log fit was capable of producing a linear relationship in the frequency of abortive colony size. Especially, the model with the parameter of *c* = 0.4 yielded the minimal AIC values at all doses (Table 4). In addition, to investigate the effects of the flexible *P_1_*, we carried out the simulation using the *P_1_* (g = 0) at all generations. There were great discrepancies at each dose (Figure 7 and Table 4). Importance of these results lies in the fact that the RCD changes at each generation.

**Table 4:** Appropriate model with the parameter of *c* judged by AIC in the abortive colonies arising from irradiated cells.

AIC				
Type	Dose (Gy)			
	0	2	4	8
*c* = 0.1	3.212	14.707	4.095	−4.868
*c* = 0.2	4.360	12.902	2.795	−7.233
*c* = 0.4	−2.608	8.190	−6.235	−11.660
fixed *P_1_*	55.177	59.600	19.338	32.353

Four different models with the parameter of *c* or fixed *P_1_* were examined, where *c* is the parameter for the simple linear model. The data of the fixed *P_1_* shows the result of the simulation using the *P_1_* (*g* = 0) at all generations.

### Estimation of Surviving Fraction by the Stochastic Branching Process Model of Abortive Colonies

**Figure 8:**
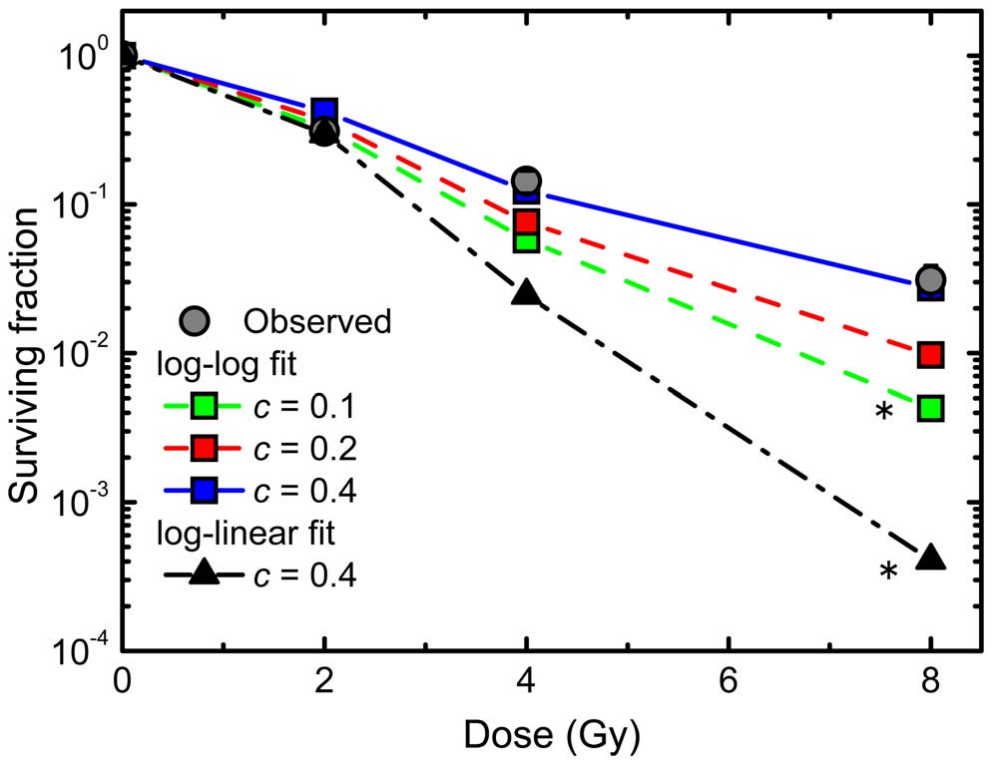
Surviving fraction estimated from the simulations of abortive colonies. Circles demonstrate the experimentally determined surviving fraction, and lines with squares represent the simulated surviving fraction with the specific slope *c* (0.1, 0.2 and 0.4) using the parameters of the log-log fit. Lines with triangles indicate the simulated ones using the parameters of the log-linear fit (*c* = 0.4). Asterisks indicate significant differences (*p*<0.05) between observation and simulation tested by the Chi square test with the Bonferroni correction.

To test a linkage between abortive and clonogenic colonies, we examined if the experimentally determined surviving fraction is consistent with that calculated from the simulated data (i.e., the number of clonogenic colonies derived from irradiated cells divided by those from non-irradiated ones). There was a significant difference in the survival curve in the log-log fit model (*c* = 0.1) and the log-linear fit model (*c* = 0.4), and this was in contrast to the case for he survival curve between the observed results and the simulated ones with the parameter of *c* = 0.2 and 0.4 (Figure 8). These results suggest that our model with the appropriate parameter of *c* can simulate the surviving fraction. Importantly, the first linkage between abortive and clonogenic colonies was established by the branching dynamic model according to the stochastic branching processes.

## Discussion

With the experimental observation and modeling approach based on the general and simple principles, we have here demonstrated that the size distribution of abortive colonies with ≥2 cells observed in the experiments did follow a linear relationship on the log-linear plot (Figure 2), but an optimal method for the colony-formation kinetics of the abortive colonies was a log-log fit in the colony size distribution, because it reflected the stochastic outcome of the branching processes. Indeed, the log-linear fit estimated a broad range of the RCD probability (Figure 5a), leading to the systematic discrepancy in the abortive colony size distribution (Figures 7a–c) and the underestimation of the surviving fraction (Figure 8). Our findings suggest that the branch system is central to production of abortive colonies, and that the present method is important as it provides a single framework to understand the behavior of primary cell colonies in culture. This encourages further studies of the model to provide a link between abortive and clonogenic colonies.

### Abortive Colony Distribution and Branching Dynamism

Abortive colony size distribution showed a linear relationship on the log-log plot (Figure 4b, 4d and 4f). It would be interesting to know whether the distribution is related to the conventional theory. The power law is well known for a linear relationship on the log-log plot, but we did not conclude that the abortive colony size distribution follows the power-law relation, because it was over only a very narrow range of colony sizes [11]. Also, the abortive colony size distribution did not follow the exponential relation, because of a steep slope between 1-cell and 2-cell colony sizes on the log-linear plot (Figure 3). On the other hand, it has been reported that *p53*-mutant clones induced at different times after ultraviolet light exposure show the power-law distribution [12]. In this case, there is a production mechanism of a power law whereby various exponential growth components of clones overlap, e.g., non-characteristic half-lives in radioactive decays [13]. Abortive colony size distribution would not include such a production mechanism. Only for *P_1_* = 0.5, the average size of colony is infinite, since all clones may eventually abort but the time taken to do so is infinite. This case comes under the birth-death process [14], and its properties are related to the power-law. However, there is not a satisfactory theoretical reason for *P_1_*>0.5 or *P_1_*<0.5. Taken together, it is still difficult to relate the production rule of abortive colony size distribution to the conventional rule or formula. Our present framework of the stochastic branching analysis could provide the probability or excess probability of RCD. The probability might give an important key to understand the production rule of abortive colonies.

### Persistent RCD

Persistent cytocidal effects in the descendants of IR-exposed cells are of important concern [5], [6], [15]. Delayed RCD persists for multiple generations after irradiation [16], [17], and its cell lineage is extremely complicated [18], [19]. In addition, the frequency of delayed RCD gradually decreases depending on the generation number [20]. On the other hand, our modeling approach also obtained similar results (Figure 5d), supportive of their experimental findings. The delayed RCD estimated by the model, i.e., excess probability, lasted over 16 generations and consisted of two components in the early (<3 generations) and late phases. Intriguingly, the excess probability at the fifth generation increased in a dose-dependent manner, whereas this was not the case for the probability at zero generation. This implies that a higher dose of IR induces more complicated and longer-lasting damage in cell division mechanisms. Indeed, the surviving fraction showed the exponential dose dependence (Figure 8).

### Abortive and Clonogenic Colonies

The conventional target theory [21] is elegant and entirely consistent with the exponential dose-response relation in clonogenic colonies, but biological mechanisms behind the target theory are still largely unknown. It might be thought that the investigation of abortive colonies is essentially the same as understanding clonogenic colonies. Indeed, our branching dynamics model for abortive colonies well simulated the dose response of clonogenic colonies (Figure 8), despite a simple assumption of *P_1_* (*g* ≥*6*). Here, it is noteworthy that, whereas the abortive colony size distribution (≤15 cells) was robust against the parameter of *c* (Figures 6a and 7a–c), the dose response curve of surviving fraction was sensitive to the excess probability over 6 generations (Figure 8). These findings strongly suggest that short-term persistent RCD is critical to the colony size distribution in small abortive colonies (≤15 cells), and that long-lasting persistent RCD is important for clonogenicity. To make further advances in our modeling study, the detailed analysis of the frequency in abortive colonies with 16–49 cells would be required, because this fraction reflects the long-lasting persistent RCD. In general, the ratio of the total number of abortive colonies can be expressed as “1– an exponential formula from the target theory”. To link or formulate the total number of abortive colonies based on the stochastic branching dynamics, i.e. formulation of the persistent RCD, is a question that should be addressed in the future.

## Supporting Information

Figure S1 **Observed and simulated growth curves.** A line with circles shows the experimentally determined growth curve, and a dashed line with squares demonstrate the simulated growth curve. Error bars indicate standard deviations.
(TIF)

Figure S2 The comparison of the simple analytical solution based on the Equation 1 and the simulation model in the size distribution of the small abortive colonies with fixed *P_1_* values.
(TIF)

Figure S3 **Computational simulation of non-irradiated abortive and clonogenic colonies using the parameters of the log-linear fit.** (a) Observed and simulated abortive colony size distribution. Gray circles show the observed colony size, and lines show the simulated colony size distribution with the slope *c* ranging from 0.0 to 1.0. A solid circle represents the estimated frequency of 1-cell colony. Several patterns of *P_1_* with the slope *c* were shown in the inset. (b) Comparison of simulated results and experimental data sets including the frequency of colonies with ≥16 cells. Error bars indicate the standard deviations. The inset demonstrates the square of the difference (SQD) between simulated and observed frequencies, calculated as Σ{(*f*_simulated_ – *f*_experimental_ )/*f*_experimental_ * 100}^2^, where *f* is the percentage of colonies with *n* cells, each of which was classified into 2–7, 8–15, 16–49 or ≥50 cells. a.u., arbitrary units.
(TIF)
