# Supplementary figures and images for: A Framework for Analysis of Abortive Colony Size Distributions Using a Model of Branching Processes in Irradiated Normal Human Fibroblasts
Source: PLoS One. 2013 Jul 23;8(7):e70291. doi: 10.1371/journal.pone.0070291 (PMC3720916; doi:10.1371/journal.pone.0070291)

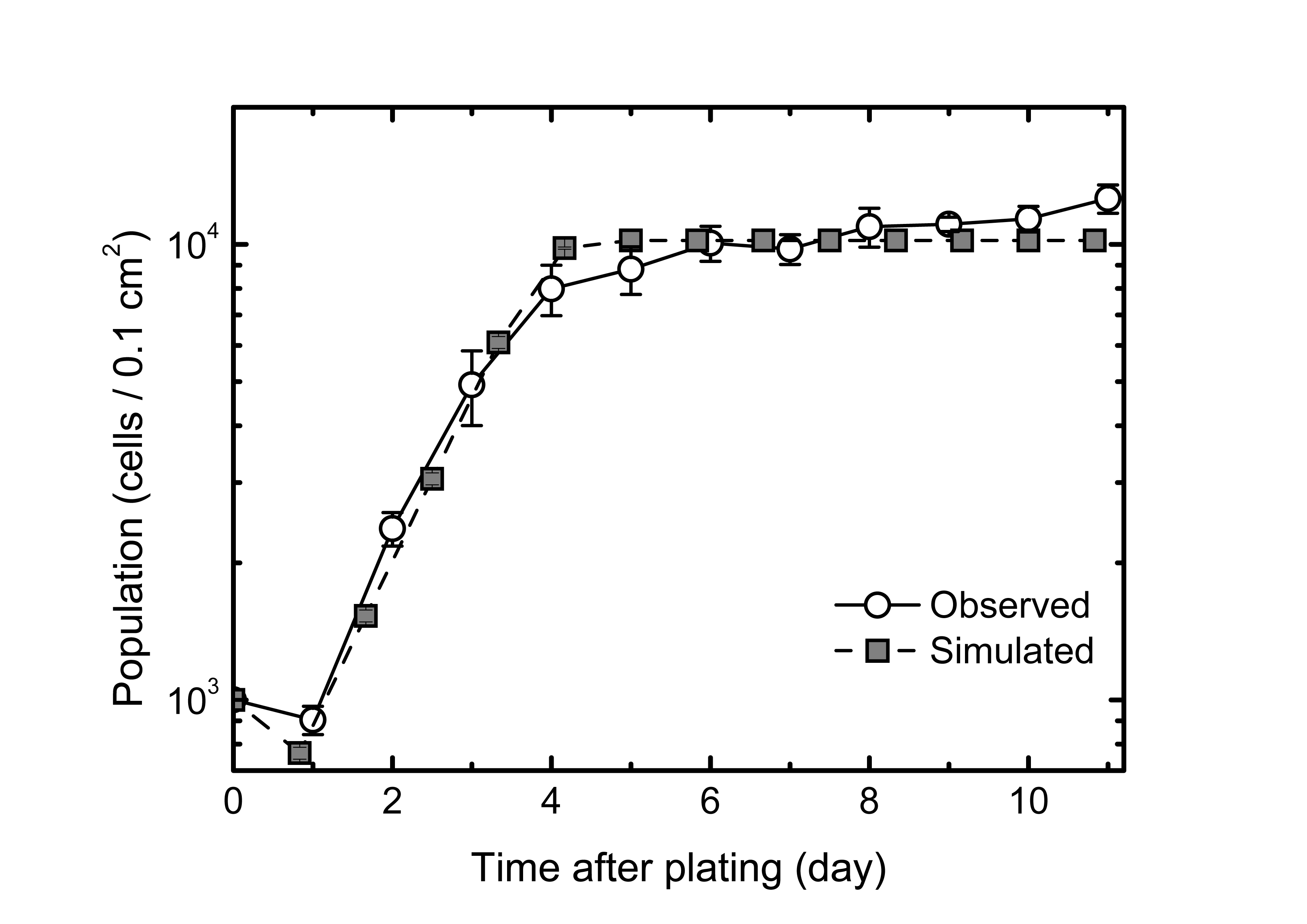

Supplement: Figure S1 — Observed and simulated growth curves. A line with circles shows the experimentally determined growth curve, and a dashed line with squares demonstrate the simulated growth curve. Error bars indicate standard deviations. (TIF) [file pone.0070291.s001.tif]

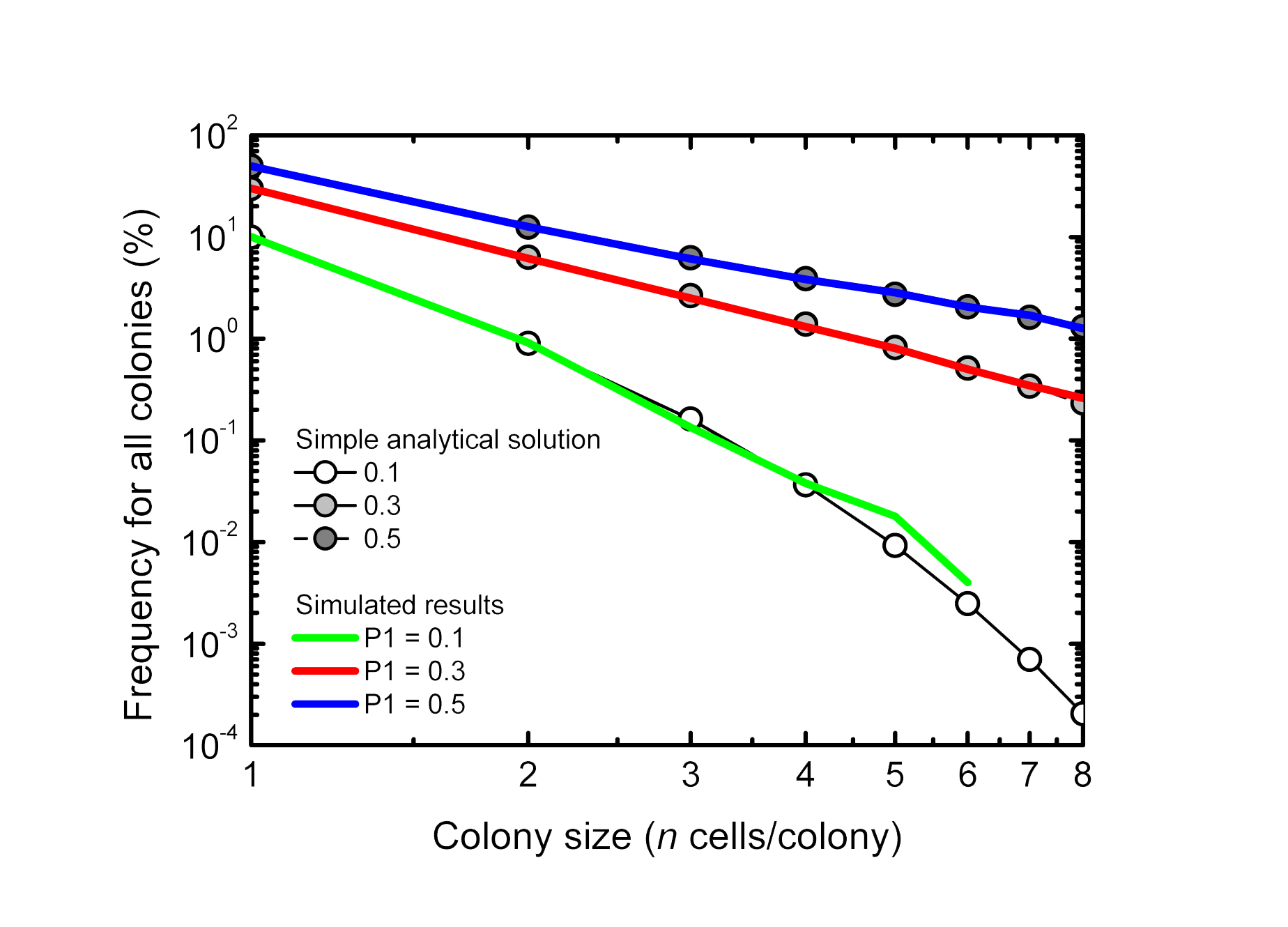

Supplement: Figure S2 — The comparison of the simple analytical solution based on the Equation 1 and the simulation model in the size distribution of the small abortive colonies with fixed P1 values. (TIF) [file pone.0070291.s002.tif]

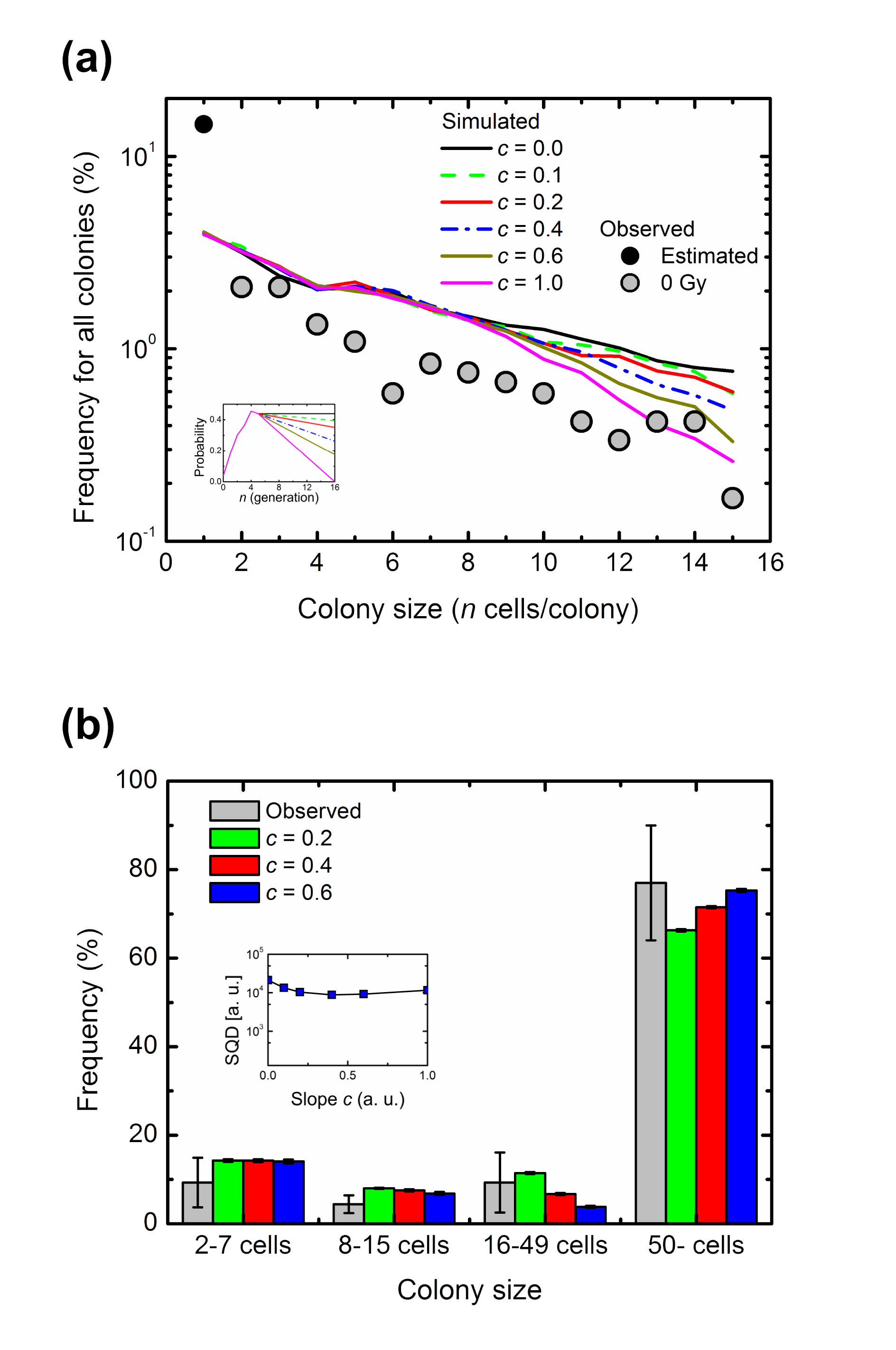

Supplement: Figure S3 — Computational simulation of non-irradiated abortive and clonogenic colonies using the parameters of the log-linear fit. (a) Observed and simulated abortive colony size distribution. Gray circles show the observed colony size, and lines show the simulated colony size distribution with the slope c ranging from 0.0 to 1.0. A solid circle represents the estimated frequency of 1-cell colony. Several patterns of P1 with the slope c were shown in the inset. (b) Comparison of simulated results and experimental data sets including the frequency of colonies with ≥16 cells. Error bars indicate the standard deviations. The inset demonstrates the square of the difference (SQD) between simulated and observed frequencies, calculated as Σ{(f simulated – f experimental )/f experimental * 100}2, where f is the percentage of colonies with n cells, each of which was classified into 2–7, 8–15, 16–49 or ≥50 cells. a.u., arbitrary units. (TIF) [file pone.0070291.s003.tif]
